# Early life immunity in the era of systems biology: understanding development and disease

**DOI:** 10.1186/s13073-018-0599-1

**Published:** 2018-11-23

**Authors:** Steven Schaffert, Purvesh Khatri

**Affiliations:** 1 Institute for Immunity, Transplantation, and Infection, Stanford University School of Medicine, Stanford, CA 94305 USA; 2 Department of Medicine, Division of Biomedical Informatics Research, Stanford University School of Medicine, Stanford, CA 94305 USA

**Keywords:** Systems immunology, Early life immunity, Immune system development

## Abstract

Systems immunology has the potential to offer invaluable insights into the development of the immune system. Two recent studies offer an in-depth view of both the dynamics of immune system development and the heritability of the levels of key immune modulators at birth.

## Development of the immune system in early life

Early life in humans (beginning at the fetal stage and progressing to the first few years of life) is associated with dramatic developmental milestones in the immune system, which makes this stage particularly important and unique. The innate branch of the immune system consists of cells such as neutrophils and macrophages, and is the first response to infection. It lacks memory and generally is activated by recognizing generic pathogen-associated molecular patterns. In contrast, the adaptive branch, consisting of cells such as B and T cells, is targeted, specific, and has memory to previously encountered stimuli. Development of the innate and adaptive branches of the immune system occurs in waves with the earliest tissue resident macrophages observed at 4 weeks of gestation and the earliest T cell development observed between 8 and 12 weeks of gestation. Compared with the adult immune system, which has incurred years of exposure to antigens and environmental stimuli, the newborn immune system emerges from a relatively sterile environment into one filled with bacterial, fungal, and viral challenges.

These differences in exposure to antigens and environmental stimuli have consequences when examining disease susceptibility. For instance, compared with adults and children, infants face increased susceptibility to infection [1, 2]. Yet many of our preventative strategies for neonates rely upon our understanding of the adult immune system because of our limited knowledge of early life immunity. To address the many outstanding questions concerning how early life environment and genetics affect the susceptibility of disease both at the early life stages and later in life requires understanding of the heritability of immune responses and the variability of the responses in a population. Studies of immune system variability in adults highlight the extensive impact the environment has on the immune response. For example, Brodin et al. [3] analyzed the heritability of immune response characteristics in twins and found that the majority of the variation cannot be explained by heritable influences, which suggests the environment plays a considerable role in shaping the adult immune response. Moreover, the variability increased with age. Similarly, in a study focusing on the epigenetics of the immune response, Cheung et al. [4] found that 70% of the inter-individual variability in chromatin modifications in immune cells was due to non-heritable factors. Both of these studies imply a model in which the immune response at early life is largely uniform across individuals and that time and its associated environmental exposures leads to divergence. Unfortunately, immunological studies on newborns tend to be small-scale and focus on only a few parameters because of limited sample volumes and low-throughput techniques. However, high dimensional single-cell technologies such as cytometry by time of flight (CyTOF) and methods to profile hundreds of plasma proteins in small volumes have made possible several new studies on early life immune system development. High-resolution understanding of the early life immune response could lead to vaccines with better efficacy in the young, help identify risk factors for autoimmunity, and improve treatment of early life infectious disease.

## Recent advances in the study of early immune system development

Two recent studies describe the first steps towards understanding early life immune system development [5, 6]. Olin et al. [5] found that early immune system development followed a stereotypic pattern in pre-term and term children, while Traglia et al. [6] compared maternal and fetal contributors to the early life immune system. Both studies made use of high-throughput technologies to measure multiple factors simultaneously.

Olin et al. [5] analyzed blood from 100 newborns: 50 pre-term births and 50 term births. They profiled cell frequencies of all major immune cell populations using CyTOF, and 267 plasma proteins by immunoassay in cord blood at birth, and in blood at weeks 1, 4, and 12 after birth. Cord blood samples were highly diverse with little correlation to the post-natal immune phenotype. Pre-term births were associated with a strong pro-inflammatory signature. Neutrophils increased with gestational age, with pre-term newborns having lower numbers compared with term newborns. Topological data analysis using both plasma proteins and immune cell population frequencies found that although children born pre-term or at term were different from each other at birth, they converged onto a stereotypical immune phenotype.

Furthermore, compared with their parents, immune systems of newborns were more dynamic over time and had much larger intra-individual variability. This is in contrast to previous work that showed that inter-individual differences were substantially larger and affected by environmental exposures [7]. In adults, repeated measures of immune system components over time remain largely stable with larger differences observed between people rather than within. Over time, phenotypes of B cells, natural killer (NK) cells, and dendritic cells (DCs) in newborns become more similar to those of their parents, whereas T cells do not. This highlights a critical developmental window for these cell types early in life. Olin et al. [5] also analyzed the microbiomes of newborns. Microbiome diversity increased after birth and those with low diversity (and high levels of activated T cell populations) exhibited increased immunological heterogeneity at 3 months of age. Furthermore, several key immune cell populations (B cells, NK cells, and DCs) reached adult-like phenotypes in the first 3 months of life, which suggests that exposures to antigens by these cells during this period could lead to diverse outcomes later in life. For example, differential susceptibility to autoimmunity and asthma may relate to DC exposure to bacterial antigens early in life, which could lead to more tolerogenic DCs later in life. Overall, Olin et al. [5] provide a detailed view of immune system development that supports a model in which the immune system is highly heterogeneous at birth but converges in the first 3 months of life.

The fetal cytokine environment has an important effect on fetal development. For example, high levels of interleukin (IL)-6, IL-1, IL-8, and tumor necrosis factor (TNF) is predictive of pre-term birth [8] and type I interferons are essential for host resistance against fetal Zika virus infection in a mouse model [9]. Traglia et al. [6] focused on the heritability of cytokine and chemokine levels between mothers and infants by conducting the first genome-wide study of immune regulators in infants and mothers simultaneously. Several cytokine and chemokine levels were measured in more than 700 mother–infant pairs and the genome-wide single nucleotide polymorphism-based heritability of each was calculated to find loci that contribute to their levels. Results from the study showed substantially less variability in cytokine and chemokine levels in infants compared with mothers. Seven chemokines had very high levels of heritability, which suggests that there is a strong genetic component to early chemotactic programs in the infant. Traglia et al. [6] identified the *PLCL2* locus as being highly associated with several inflammatory cytokines and chemokines that are implicated in the humoral response and B cell receptor signaling (interferon γ, IL-2, chemokine c-c motif ligand 7, chemokine c-x-c motif ligand 9, and chemokine c-c motif ligand 19).

Maternal genetics contributed substantially to the levels of six cytokines or chemokines in the infant [6]. Of these, the neonatal level of the cytokine IL-4 was not influenced by fetal genetics but only by maternal heritability. Intriguingly, fetal genetics did contribute to the levels of cytokines and chemokines in mothers. For instance, a fetal single nucleotide polymorphism within a long noncoding RNA near *ADCYAP1* was associated with maternal soluble IL-2 receptor alpha (sIL-2Ra) chain levels. Fetal genetics contributed to the maternal cytokine milieu likely through signaling at the placenta, not through direct transfer of cytokines and chemokines across the placenta. Certain cytokines in the newborn can be explained by maternal genotype, and sIL-2Ra in mothers can be explained by the newborn genotype, which suggests a bi-directional interaction. This analysis of cross heritability begins to shed light on the complex dynamics of maternal–fetal immunological interactions.

Both of these studies look at early life immune system development using high-throughput technologies. While they approach this question from different angles, taken together they construct a framework for understanding the heritable and environmental factors that characterize early life immunity.

## The way forward

The findings presented in both studies have implications for long-term health and disease. The unique critical developmental time windows for immune cell types suggest that exposures at specific times could imprint themselves onto these subpopulations, which could have consequences for the immune response later in life. As one example, an early pro-inflammatory environment could inhibit peripheral T cell tolerance to insulin, which could lead to type I diabetes. Vaccination in neonates also does not behave as it does in older individuals. With the insights from Olin et al. [5], vaccinologists may be able to develop better vaccine schedules that focus on the B cell development critical window.

These studies provide an example of the unique insights that can be gained from a systems-level approach to immunology. Olin et al. [5] used high dimensional cytometry to compute an immunological distance between parents and newborns by calculating distributional distances based on multiple phenotypic markers, which moves beyond simply looking at frequencies of cells. Results from these studies also pave the way for the next set of important questions which can be addressed using systems immunology approaches. For instance, a similar study of the T and B cell receptor repertoires in early life could shed light on how the adaptive response develops over time in response to environmental exposures. Analogous to the study by Olin et al. [5], it would be useful to examine the convergence or divergence of other repertoires from pre-term and term births. Extending the observational timeline of these studies, following up later in life to look for correlates of early immune features with disease outcomes later would also be insightful. Although environmental exposure is shown to increase cell-to-cell variability in the epigenome of the adult immune system [4, 10], characterization of the epigenome in immune cells at birth and during immune development in early life is lacking. Results from Olin et al. [5] suggest that a critical developmental window for certain cell types could make environmental exposures during this window particularly important for disease susceptibility later in life. Understanding how imprinting via epigenetics at this stage is associated with disease risk later in life will be extremely useful.

## Abbreviations

CyTOF: Cytometry by time of flight
DC: Dendritic cell
IL: Interleukin
NK: Natural killer
sIL-2Ra: Soluble interleukin-2 receptor alpha
